# Various Techniques for the Surgical Treatment of Common Bile Duct Stones: A Meta Review

**DOI:** 10.1155/2009/840208

**Published:** 2009-08-06

**Authors:** Abolfazl Shojaiefard, Majid Esmaeilzadeh, Ali Ghafouri, Arianeb Mehrabi

**Affiliations:** ^1^Department of Surgery, Shariati Hospital, Tehran University of Medical Sciences, Tehran, Iran; ^2^Department of General, Visceral and Transplantation Surgery, University of Heidelberg, 69120 Heidelberg, Germany

## Abstract

Common bile duct stones (CBDSs) may occur in up to 3%–14.7% of all patients for whom cholecystectomy is preformed. Patients presenting with CBDS have symptoms including: biliary colic, jaundice, cholangitis, pancreatitis or may be asymptomatic. It is important to distinguish between primary and secondary stones, because the treatment approach varies. Stones found before, during, and after cholecystectomy had also differing treatments. Different methods have been used for the treatment of CBDS but the suitable therapy depends on conditions such as patient' satisfaction, number and size of stones, and the surgeons experience in laparoscopy. Endoscopic retrograde cholangiopancreatography with or without endoscopic biliary sphincterotomy, laparoscopic CBD exploration (transcystic or transcholedochal), or laparotomy with CBD exploration (by T-tube, C-tube insertion, or primary closure) are the most commonly used methods managing CBDS. We will review the pathophysiology of CBDS, diagnosis, and different techniques of treatment with especial focus on the various surgical modalities.

## 1. Introduction

CBDSs are one of the medical conditions leading to surgical intervention. They may occur in 3%–14.7% of all patients for whom cholecystectomies are preformed [1, 2]. When patients present with CBD, the one important question that should be answered: what is the best modality of treatment under the giving conditions? There are competing technologies and approaches for diagnosing CBDS with regard to diagnostic performance characteristics, technical success, safety, and cost effectiveness. Management of CBDS usually requires two separate teams: the gastroenterologist and the surgical team [3]. One of the main factors in the management is initially the detection of CBDS, before, during, or after cholecystectomy. The main options for treatment are pre- or postoperative ERCP with endoscopic biliary sphincterotomy (EST), laparoscopic or open surgical bile duct clearance. There are other options for the treatment of CBDS such as electrohydraulic lithotripsy (EHL), extracorporeal shockwave lithotripsy (ESWL), dissolving solutions, and laser lithotripsy. It is unlikely that one option will be appropriate for all clinical circumstances in all centers. Variables such as disease status, patient demographics, availability of endoscopic, radiological and surgical expertise, and healthcare economics will all have significant influence on practice [4].

## 2. Method

**Figure 1:**
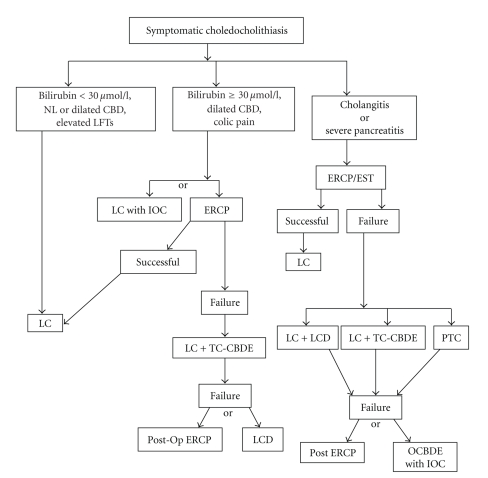
Algorithm for management of common bile duct stones. LC: Laparoscopic cholecystectomy, LCD: Laparoscopic choledochotomy, PTC: Percutaneous transhepatic catheter drainage, TC-CBDE: Transcystic common bile duct exploration, OCBDE: Open common bile duct exploration, IOC: Intraoperative cholangiogram.

A Medline-based search on all published papers (English and German) for CBDS diagnosis and treatment was performed. The search terms used for the review included common duct stones, clinical presentation of CBDS, diagnostic approach of CBDS, MRCP, transabdominal ultrasonography, intraoperative cholangiography, common duct exploration, common bile duct exploration, laparoscopic common bile duct stone endoscopic sphincterotomy, trans-cystic, and ductal approach. This paper serves to delineate the current relevant concepts in the varying treatments of patients that present with CBDS. We also present a possible algorithm for the treatment of CBDS (Figure 1).

## 3. Pathogenesis and Clinical Manifestation

CBDS can be caused either by primary bile duct stones that originate in the bile duct or by secondary bile duct stones that have descended from the gallbladder [6]. In the primary stones, bilirubin is dominant component and is associated with biliary stasis and infection. In secondary stones, cholesterol is dominant component. It is therefore important to distinguish between primary and secondary stones. Cholecystectomy and choledocholithotomy are sufficient in the management of secondary stones, while the presence of primary stones often necessitates a more complex drainage procedure to prevent recurrence [7, 8]. Table 1 shows the types of bile duct stones [5]. In addition, cholecystectomy at a young age leads to CBD dilatation and is another acquired risk factor for CBD stones [9].

The symptoms and signs of CBDS are highly variable and can range from patients being completely asymptomatic, to complications such as cholangitis or pancreatitis [10]. Literature describes the Prevalence of asymptomatic CBDS between 5.2% and 12% [11]. A common presentation of CBDS is the biliary colic. Pain is often situated in the right hypochondrium or epigastrium and can last from 30 minutes to several hours, with associated symptoms such as nausea and vomiting [10]. Other common symptoms include pale stools and dark-colored urine, which can be elicited in the patient history by a thorough review of systems [12]. Two serious complications of CBDS are cholangitis and gallstone pancreatitis. Acute obstructive cholangitis (AOC) is a life-threatening complication caused by an infection of the biliary ductal system secondary to biliary obstruction. Cultures are most often positive for *E. coli*, and the infection clears in more than 75% of cases with antibiotic treatment [13]. In cholangitis, the classic symptoms of Charcot's triad may be encountered, and the less common Reynold's pentad adds to the diagnosis [7, 13]. Despite the advancement in treatment, AOC still carries a mortality rate of 10–20% [14].

It remained unclear for a long time why some gallstone patients suffer from pancreatitis, while others are spared from this potentially lethal complication. Recent data indicate that small gallstones, excess cholesterol crystals, and good gallbladder emptying are associated with increased risk of pancreatitis [15, 16]. Small gall stones could lead to a more distal obstruction with potential reflux of bile into the pancreatic ducts. This could induce a common pathway of pancreatic duct injury with release of activated pancreatic enzymes into the glandular interstitium [17]. The majority of these patients will have self-limiting disease, but mortality still remains about 10% [18]. The mortality rate is less than 1% for mild acute pancreatitis, but it can approach 10% to 30% for severe acute pancreatitis [19].

## 4. Assessment and Diagnosis

### 4.1. Laboratory Tests

Patients exhibiting the described symptoms require diagnostic investigation to assess for the presence of CBDS [12]. Liver function tests (LFTs) can be used to screen for CBDS [20, 21]. Elevated serum bilirubin and alkaline phosphatase typically reflect biliary obstruction, but these are neither highly sensitive nor specific for CBDS [22]. In a study by Anciaux et al., elevated serum gamma glutamyl transpeptidase (GGT) and alkaline phosphatase (ALP) were the most frequent abnormalities in laboratory valves of patients with symptomatic CBDS [10]. Serum bilirubin levels may be markedly elevated depending on whether the obstruction of the bile duct is complete or incomplete [10]. Murohisa et al. [23] and Sheen-Chen et al. [24] in one case study reported high level CA 19-9 in CBDS with cholangitis. Most of the studies have shown that laboratory studies must be used in addition to imaging modalities to predict the likelihood of CBDS, and the multivariate analysis models have found a dilated bile duct to be an independent variable in predicting CDBS [25–27].

### 4.2. Imaging Modalities

#### 4.2.1. Transabdominal Ultrasonography (TUS)

It is the first line investigation in patients with suspected CBDS [10]. Its sensitivity for detecting CBDS is between 25% to 63% [28], with a specificity of approximately 95% [28] depending on the degree of dilation of the CBD and investigators experience. Barkun et al. reported that in patients older than 55 years with abnormal liver enzymes and CBD dilation in ultrasound examination, CBDS is predicted in up to 95% [29]. Endoscopic retrograde cholangiopancreatography (ERCP) is often described as the gold standard test to for the detection of CBDS [10]. This procedure was initially used primarily in diagnosis, but today is more commonly used as a therapeutic modality [12]. ERCP has sensitivity between 90% to 95% in detecting CBD stones [30, 31] and a specificity of 92% to 98% [32, 33]. Christensen et al. demonstrated that the ERCP exam has a morbidity rate of 15.9% and a mortality rate of 1% [34].

#### 4.2.2. Endoscopic Ultrasound (EUS)

It involves the endoscopic insertion of an ultrasound probe through the stomach and up to the second half of the duodenum, allowing for ultrasound images of the CBD without the interference of subcutaneous fat and bowel gas [35]. Sensitivity of EUS varies from 95%, while specificity is between 95–98% [36]. EUS is significantly more sensitive than TUS in detecting CBD stones. Its sensitivity is comparable to the diagnostic ERCP, while its major advantage is a significantly decreased morbidity compared to ERCP [12, 37]. The EUS exam is a noninvasive test, with excellent overall sensitivity and specificity for diagnosing choledocholithiasis, but it is highly dependent on the examiner.

#### 4.2.3. Magnetic Resonance Cholangiopancreatography (MRCP)

It has emerged as an accurate, noninvasive diagnostic modality for investigating the biliary ducts [22, 38]. It may be especially beneficial in identifying patients who would benefit from early intervention [12, 39–41]. A recent authoritative meta-analysis of 67 published controlled trials shows that MRCP has an excellent overall sensitivity of 95% and a specificity of 97% for demonstrating CBDS [42–44]. Verma et al. reported no statistically significant differences between EUS and MRCP in the detection rate of CBDS [45]. Some major disadvantages of MRCP, as compared to ERCP, are the lower spatial resolution [46], unit availability, potential for claustrophobia, and the inability to evaluate patients with pacemakers or ferromagnetic implants [42].

#### 4.2.4. Intraoperative Cholangiography (IOC)

The routine use of IOC is still controversial. Some authors supporting routine IOC [47, 48], while others favor selective IOC [49, 50], and others report no advantages in IOC [51–53] with respect to missed CBD stones. However, it can be an useful tool to identify choledochal stones [22]. This procedure can be performed during open or laparoscopic cholecystectomy. IOC has a sensitivity of 98% and specificity of 94% to detection of CBDS [54]. IOC can fail primarily due to inability to cannulate the cystic duct. Other reasons for failure are leakage of contrast fluid during the injection, air bubbles mimicking stones, failure to fill the biliary tree because of too rapid contrast injection into the duodenum, and spasm of the sphincter of Oddi. Supporters of routine IOC claim that this practice ensures fewer retained stones, fewer postoperative ERCPs, and a reduction in the number of CBD injuries [55, 56]. One drawback is the consequent lengthening of the operative time by approximately 15 minutes [33, 57].

#### 4.2.5. Conventional Computed Tomography (CT)

It has a sensitivity of 87% and a specificity of 97% for the diagnosis of CBD stones [58–60]. Kondo et al. showed that CT scanning was equivalent to MRCP [61], with the added risk of allergic reaction to contrast injection [62].

#### 4.2.6. Intraductal Ultrasonography (IDUS)

Although the utility of intraductal ultrasonography (IDUS) for common bile duct stones has been reported, the clinical significance of this procedure in making therapeutic decisions has not been well clarified [63]. IDUS is a valuable method for residual small stones in the common bile duct after endoscopic lithotripsy [64]. IDUS increases sensitivity and specificity in the diagnosis of choledocholithiasis, and these gains are not coupled with a notable increase in procedure time (7–15 minutes) [65]. IDUS is especially recommended in patients who have a dilated bile duct with suspected small bile duct stones when ERCP is not diagnostic [64].

#### 4.2.7. Percutaneous Transhepatic Cholangiography (PTC)

It is not a routine initial diagnostic test in patients with CBD stones [42] but is the modality of choice in patients with previous gastric surgery, distal obstructing CBDS that failed ERCP or in patients with cholangiohepatitis and extensive intrahepatic stone disease. It is important to consider that uncorrected coagulopathy is a contraindication for PTC.

## 5. Treatment

### 5.1. Medical

Patients with cholangitis or gallstone pancreatitis are generally acutely ill, and they often require aggressive rehydration as well as complete bowel rest [12]. Enteric gram-negative bacteria are usually cultured from the bile of patients with acute cholangitis, especially E. coli and Klebsiella species. In the last decades the microbiological profile has changed due to increased instrumentation of the bile ducts and wide spread use of antibiotics in the population. Polymicrobial bile cultures are also often found. Anaerobic bacteria are usually isolated in conjunction with aerobic bacteria [66]. Choice of antibiotics should be influenced by patient characteristics (e.g., antibiotic hypersensitivity, renal function, hearing loss, severity of disease, previous instrumentation of the bile ducts) and regional antibiotic sensitivity patterns [66]. The combination of an aminoglycoside with amoxicillin-clavulanic acid is primarily used as the first-line of treatment [66]. In the event of contraindications to aminoglycosides, broad-spectrum penicillin (e.g., piperacillin or piperacillin-tazobactam) is a reasonable alternative.

### 5.2. Intervention or Surgery

Today, therapeutic decision-making is based on the local availability of expertise. Two groups of interventions have significant roles in management of CBD stones (1) pre- or postoperative ERCP with endoscopic biliary sphincterotomy (EST) in a *two-stage procedure*, (2) surgical bile duct clearance and cholecystectomy as *one-stage procedure*. Several randomized controlled trials showed similar effectiveness for both methods of treatment [67, 68]. Kharbutli et al. reported that one-stage management of symptomatic CBDS is associated with less morbidity and mortality (7% and 0.19%) than two-stage management (13.5% and 0.5%) [69]. Other methods include electrohydraulic lithotripsy (EHL), extracorporeal shockwave lithotripsy (ESWL), laser lithotripsy, and dissolving solutions that are indicated only in special situations. Although these techniques are useful in the management of the complicated biliary tract, they are not without cost, morbidity, mortality, and significant reduction in quality of life [70].

#### 5.2.1. Preoperative Endoscopic Management

More than a decade ago, randomized controlled trials showed superior outcomes for standard open bile duct surgery as compared to the endoscopic (ERCP/EST) treatment of CBDS [71]. ERCP/EST was performed with leaving the gallbladder in situ in patients with preoperative cholangitis or pancreatitis, older than 80 years of age, substantial comorbidity and where CBD stones were discovered. Although the success rate for stone clearance in isolated ERCP treatment is up to 87% to 97%, up to 25% of patients require two or more ERCP treatment [72]. This method is associated with morbidity and mortality rates of 5% to 11% and 0.7% to 1.2%, respectively [73, 74]. Schreurs et al. showed 75%–84% patients undergoing ERCP/EST had no symptoms with up to 70-month followup [75]. Complications of ERCP include bleeding, duodenal perforation, cholangitis, pancreatitis, and bile duct injury [76]. Moreover, ERCP is not possible in 3% to 10% of all patients [77].

Endoscopic balloon dilation of the papilla has been advocated as an alternative method to EST, in comparison to this procedure is easier [78], has lower bleeding rate [79, 80], less disruption of function to the sphincter of Oddi [81]. In comparison to EST, the rate of pancreatitis is higher than EST and is not the procedure of choice for patients undergoing stone extraction [82]. Weinberg et al. reviewed several randomised clinical trials comparing endoscopic balloon dilation versus EST for the removal of CBDS and reported that endoscopic balloon dilation is less successful than EST. In these cases, endoscopic balloon dilation was done, respectively, in patients with coagulopathy, and at risk for infection [83].

It is important to ensure adequate biliary drainage in patients with CBDS that have stoned not yet extracted. Therefore, short-term use of a biliary stent followed by further endoscopy or surgical treatment is advocated [84]. For patients over 70 years of age or with debilitating disease, biliary stenting has also been examined as an alternative to the endoscopic method [85]. Biliary stenting as a “bridge” to further therapy is recommended, as is its use as a definitive treatment for CBDS should be restricted to patients who have limited life expectancy or are judged by a surgeon to be at prohibitive surgical risk [84].

#### 5.2.2. Laparoscopic Common Bile Duct Exploration

The successful laparoscopic management of CBD stones depends on several factors including surgical expertise, adequate equipment, the biliary anatomy, and the number and size of CBD stones [86]. With advancing technology and minimally invasive surgery, laparoscopic biliary surgery has become safe, efficient, and cost effective [87–89]. Laparoscopic common bile duct exploration (LCBDE) was associated with successful stone clearance rates ranging from 85% to 95%, a morbidity rate of 4%–16% and a mortality rate of around 0%–2% [90, 91]. Laparoscopic exploration is very effective for clearing difficult CBD stones. Tai et al. reported that the clearance rate was 100%, and no recurrence was discovered during a mean followup period of 16 months [76]. Golipour et al. showed LCBDE to be an effective procedure as the initial modality of management for acute gallstone cholangitis [92]. Complications from this method include CBD laceration, stricture formation and bile leak [93]. Patients treated with LCBDE had a significantly shorter hospital stay and lower hospital costs as compared with ERCP/EST [88].

During laparoscopic cholecystectomy, if CBDSs are found with IDUS, IOC, or other modalities, LCBDE can be performed. There are two primary methods for LCBDE: trans-cystic (via the cystic duct) and trans-ductal (via choledochotomy). If CBDS are detected at the time of laparoscopic cholecystectomy, the best treatment is a trans-cystic laparoscopic approach during the same operation. If this fails, alternate approaches such as intraoperative or postoperative ERCP/EST, laparoscopic choledochotomy, or open CBDE may be used [94]. A trans-cystic approach is generally used for small stones in a small bile duct whereas trans-ductal approach is preferred for large occluding stones in a large duct, intrahepatic stones, or a miniscule or tortuous cystic duct [95]. Selection of the differing approaches is influenced by several factors [70] (Table 2).

**LCBDE-Trans-Cystic Approach**

In the trans-cystic approach, 100–200 mL isotonic sodium chloride solution with 1–2 mg glucagon (for relaxation of Oddi's sphincter) is used to irrigate the CBD in an attempt to flush small stones through the sphincter of Oddi or out through the opening in the cystic duct. If this is not successful, a helical basket can be passed over a guide wire through the cystic duct to extract stones under fluoroscopic guidance [96]. Today, LCBDE under fluoroscopic guidance seems to be the procedure of choice. If this procedure fails, a choledochoscope (≤10 Fr) should be subsequently attempted in order to remove the stones under direct sight [96]. There is only little data regarding the use of choledochoscopy in the treatment of CBDS [97]. If the CBD stone is larger than the lumen of the cystic duct, the cystic duct can be balloon-dilated. This dilation should never be larger than the internal diameter of the CBD [98]. A flexible choledochoscope can be passed into the peritoneal cavity through a midaxillary port and the CBD examined under direct sight. The CBD should be kept inflated with isotonic sodium chloride solution for better visualization. Intraluminal stones can be extracted with a basket under direct vision using the working port of the scope. A Segura-type stone basket is advanced via the working channel of the scope beyond the stone and then opened. As the basket is pulled backwards and simultaneously rotated, the stone is ensnared [99]. A cholangiogram or ultrasound should always be performed to conclusively demonstrate clearance of the duct [96]. The outcome of the transcystic method proved to be consistent with the goals of a laparoscopic approach: minimal morbidity, no T-tube, no drain, and a rapid return to normal activity in most cases [70].

Other novel transcystic approaches include balloon dilatation of the sphincter of Oddi and antegrade sphincterotomy. Balloon dilatation of the sphincter of Oddi can be performed when all other techniques have failed to clear the stones. A risk exists for developing mild pancreatitis with this method (15% in one series) [100]. Therefore, this technique should be avoided in patients with pre-existing pancreatitis, CBD dyskinesia, or sphincter anomalies. Successful transcystic duct clearance has been described in 80%–98% of patients in a recent series [101, 102]. Complications such as infection and pancreatitis have been reported in 5%–10% of patients, with a mortality rate of 0%–2%. The duration of hospitalization following an uncomplicated transcystic duct stone extraction is the same as that for laparoscopic cholecystectomy alone, averaging approximately 1–2 days. The main advantage of the transcystic approach is that it avoids the need for choledochotomy [96].

**LCBDE-Trans-Ductal Approach**

If the transcystic approach fails, we recommend laparoscopic choledocholithotomy. Laparoscopic choledocholithotomy can be accomplished with a variety of techniques. Choledocholithotomy may involve performing a number of technical maneuvers such as dilation of the distal CBD, balloon catheter manipulation, basket manipulation with or without fluoroscopic guidance, choledochoscopic manipulations [100, 103] as well as IOC. After the stones are removed under endoscopic visualization, the ductotomy is usually closed either primarily or over an appropriately sized T-tube. The indication for T-tube insertion is decompression of the duct in patients with residual distal obstruction, ductal imaging in the postoperative period and providing an access route for the removal of residual CBD stones [70]. Most authors prefer a longitudinal choledochotomy over a distance of approximately 1–1.5 cm, a 14-French latex T-tube (or larger), and closed over a 16-French T-tube using 4-0 monofilament absorbable sutures. Some centers use transcystic tubes (C-tube) or antegrade stenting with choledochorrhaphy for CBD drainage [104, 105]. C-tube drainage via the cystic duct following CBD exploration would seem to be the preferred choice of treatment for patients who require choledochotomy because of large multiple stones in the CBD using this technique [106].

Management of T-tubes in the postoperative period may involve bacteremia, dislodgment of the tube, obstruction by the tube, or fracture of the tube [107]. Broad-spectrum antibiotic coverage while the T-tube is in situ may be necessary. The patient can generally be discharged 2–4 days postoperatively. T-tube cholangiography should be performed before removal of the tube (6–18 days postoperatively). Removal of T-tubes has been suggested as early as 5–6 days postoperatively and as late as 4–5 weeks after surgery. Retained stones demonstrated by T-tube cholangiography may be effectively removed percutaneously after allowing maturation of the T-tube tract. Percutaneous extraction is successful in more than 95% of patients with retained stones, otherwise postoperative ERCP can be required [96]. Despite the advantages of T-tube drainage and because of the potential complications of T-tube placement, primary closure of the CBD without drainage has been advocated by some authors in open biliary tract surgery [108]. Shorter operative times and lengths of hospital stay have been observed with primary closure. No increase in bile leakage or peritonitis has been noted with primary closure in the open literature. Higher patient satisfaction has also been associated with primary closure [70]. Some studies proposed that choledochotomy with primary laparoscopic closure of the CBD is safe, eliminates the need for T-tube placement, and reduces operating time and postoperative morbidity [109, 110]. Yamazaki et al. reported significant differences in hospital stay between primary closure and T-tube insertion (18.3 days versus 31.5 days) [111]. In other study, Leida et al. showed in patient with primary closure of the CBD that the time until return to work (12.6 ± 5.1 versus 20.4 ± 13.2 days) was significantly shorter. Hospital expenses were significantly lower and the incidence of postoperative complications (15% versus 27.5%) and specially biliary complications (10% versus 20%) were significantly lower than in the T-tube drainage patients [112].

#### 5.2.3. Postoperative Evaluation and Management

Postoperative ERCP is used as a treatment modality for CBDS clearance when LCBDE failed or retained stones are discovered after an operation (2.5%) [113]. If secondary ERCP fails, clinicians must be ready for laparoscopic or open exploration. Percutaneous transhepatic therapies can be considered for CBDS under TUS guidance in selected patients [114]. Extraction of stones, sphincterotomy, or percutaneous drainage can be performed using this method [115, 116]. New approaches have been performed which exclude ERCP, such as past gastric surgery. The most common gastric surgery presently performed is the Roux-Y gastric bypass. In which, a small gastric pouch is created and anastomosed to a limb of the jejunum [114]. The majority of the stomach, duodenum, and proximal jejunum are bypassed by this method. Combined laparoscopic surgical and endoscopic procedures have also been described. Endoscopic access can be achieved via a gastrostomy or jejunostomy [117, 118]. The endoscope can also be passed into the abdomen during surgical management, and an ERCP can be performed in the standard technique. These procedures have been described in few case reports [114].

#### 5.2.4. Open Common Bile Duct Exploration

When LCBDS and postoperative ERCP have failed, the surgeon must use the open approach to surgery. Martin et al. reported open surgery as being more successful and being lower mortality than ERCP in CBDS [119]. There are two options for open common bile duct exploration: choledochoenterostomy or a sphincterotomy. Surgeon experience should therefore dictate which one is performed [120]. Some authors prefer choledochoenterostomy for CBD greater than 2 cm in diameter in order to create a large opening between the bile duct and intestine.

*Sphincterotomy* consists of incising the distal part of the sphincter musculature over a length of approximately 1 cm. This incision should not extend beyond the outer wall of the duodenum [96]. After the choledochotomy, a catheter or dilator is passed distally and a Kocher maneuver is performed, then duodenotomy is performed at the level of the ampulla. The dilator is advocated to bring the ampulla into the operative field, where it is then incised sufficiently along the anterosuperior border (opposite the pancreatic duct orifice) to permit removal of the impacted calculus [96].

*Choledocoenterostomy* is the most commonly performed as a side-to-side choledochoduodenostomy, usually in the setting of a dilated CBD with multiple stones [96], a recurrence of CBDS in the Vater's papilla occurred after ES and dilated CBD (≥2.0 cm). These patients require drainage for good long-term results without recurrence of jaundice or cholangitis [121]. The technique most commonly used is that of a side-to-side hand-sutured anastomosis between the supraduodenal common bile duct and the duodenum [122]. A Kocher maneuver is performed and the distal CBD is exposed. Choledochotomy is made within 2-3 cm of the lateral border of the duodenum. A diamond-shaped anastomosis is performed with interrupted absorbable sutures. One potential complication is the “sump syndrome” caused by food or other debris caught in the distal CBD [123]. This complication is rare (1%) and can be managed with ERC/ES [124]. The alternative operation, transection choledochoduodenostomy, excludes the distal (transpancreatic) segment of the bile duct from the end-to-side anastomosis of the transected common bile duct with the second part of the duodenum. The long-term results of this procedure are excellent [122]. Another optimal option is the choledochojejunostomy with a roux-en-Y loop.

#### 5.2.5. Electrohydraulic Lithotripsy (EHL)

EHL uses direct high voltage to generate a shockwave through a liquid medium to fragment the bile duct stone. The procedure has been performed successfully under cholangioscopic guidance [10, 125] or under fluoroscopic control using a balloon catheter [126]. Typically, its use is reserved for cases of CBD packed with multiple faceted stones or a single large impacted stone. For EHL to be successful the stone must be targeted under direct sight, otherwise there is increased risk of damaging the bile duct wall [127]. This method is rarely used because of its high potential for tissue damage and bleeding.

#### 5.2.6. Extracorporeal Shockwave Lithotripsy (ESWL)

ESWL was first used treating gallstones in 1980s following its successful use in fragmenting renal calculi [10]. ESWL involves the percutaneous administration of sound waves directed at the liver and bile duct. It is not performed during endoscopy, but rather before an ERCP in hopes of shattering large stones into smaller, more manageable fragments [127]. European studies evaluating ESWL report duct clearance rates of 83% to 90%, but its acceptance in the United States has been slow [127, 128].

#### 5.2.7. Laser Lithotripsy

Laser lithotripsy uses amplified light energy at a particular wavelength, which is focused into a single beam and directed onto a stone within the bile duct [10]. Laser lithotripsy can be performed under direct vision with cholangioscopy using mini scopes or can be performed under fluoroscopic control using standard equipment [10]. The success rate of duct clearance for retained CBDS using laser lithotripsy is between 64% and 97% in several studies [129].

#### 5.2.8. Dissolving Solutions

Table 3 shows several types of solutions that are used for dissolving gallstones and CBD stones. These solutions have few toxic side effects and do not cause irritation of the biliary tree. Every dissolution therapy will last for several weeks, therefore the ideal solvent has not yet been produced [5]. The use of ursodeoxycholic acid (UDCA) and chenodeoxycholic acid has only been shown to dissolve cholesterol-containing stones. Approximately 85–95% of patients in the Western World will have cholesterol stones. Continuing therapy with UDCA appeared to prevent recurrence of gallbladder microlithiasis [130]. Methyl-Tert-butyl-Ether (MTBE) is an excellent cholesterol solvent that has been shown to work faster, but it is toxic to liver and duodenal mucosa. It has been proposed by several studies that using dissolution in combination with endoscopic retrieval or lithotripsy has better outcomes [5, 131, 132]. Katsinelos et al. suggested that UDCA does not seem to contribute to the reduction in stones' size or stones' fragmentation during the endoprosthetic procedure [133].

Recurrence of CBD stones after ES is reported in a considerable number of patients (6–21%), resulting from de novo primary stone formation or recurrent secondary migration from the gallbladder [134]. Primary CBDSs are associated with bactobilia and delayed bile-duct clearance which is indicated by CBD dilation. Endoscopic reintervention is safe and usually easy to perform. Surgery should only be reserved for intractable cases. In selected patients, an underlying lithogenic bile composition (low-phospholipid-associated cholelithiasis) should be identified and preventive medical treatment with UDCA can be considered [134].

## 6. Conclusion

Today, management of CBDS is a complicated procedure for the treating medical stone. Ultrasonography and ERCP are routine diagnostic modalities in most centers, but clinicians can often choose from other low-invasive modalities such as MRCP or CT. LCBDE (trans-cystic or trans-ductal) is a standard method with a high efficacy and low morbidity and mortality for the treatment of CBDS in most centers. Pre- or postoperative ERCP/EST can be use as an alternative method. We recommend that for patients with CBDS, ERCP should be performed as a first step and in the event of failure LCBDE can be performed. It should not be forgot that the open approach always remains as a final option when others modalities have failed. Electrohydraulic lithotripsy, extracorporeal shockwave lithotripsy, laser lithotripsy, and dissolving solutions have especial indications and more clinical trial in this area must be performed.

## Figures and Tables

**Table 1:** Classification of gallstones [5].

	Cholesterol	Brown-pigment stone	Black-pigment stone
Origin	Gallbladder (secondary stones)	Ducts ± gallbladder (primary stones)	Gallbladder ± ducts (primary or secondary stones)
Component	40–70% cholesterol	15% cholesterol	2% cholesterol
		60% calcium bilirubinate	6% calcium carbonate
		15% calcium phosphate	40% calcium bilirubinate
			9% calcium phosphate
Predisposing factor	–Obesity	–Diet: low protein, high carbohydrate	–Cirrhosis
	–↓Bile duct pool	–Cholangitis	–Chronic hemolysis
	–↑Cholesterol synthesis	–Biliary stricture	–Sickle cell anemia
	–↑Progesterone	–Biliary infections: bacterial, parasitic	–Heart valve replacement
		–Biliary stasis: total parenteral nutrition, vagotomy	
Shape, size, number	Multiple: smooth faceted	Smooth, round	Multiple, irregular, or smooth
	Single: ≥2.5 cm, smooth, round	1–3 cm	usually <0.5 cm
Physical characteristics	Hard, laminated	Hard	Soft, friable

**Table 2:** Effective and important factors in CBD stones approach [70].

Factor	Trans-cystic approach	Trans-ductal approach
Single stone	Yes	Yes
Multiple stones	Yes	Yes
Stones < 6 mm diameter each	Yes	Yes
Stones > 6 mm diameter each	No	Yes
Intrahepatic stones	No	Yes
Diameter of cystic duct < 4 mm	No	Yes
Diameter of cystic duct > 4 mm	Yes	Yes
Diameter of common bile duct < 6 mm	Yes	No
Diameter of common bile duct > 6 mm	Yes	Yes
Cystic duct entrance—lateral	Yes	Yes
Cystic duct entrance—posterior	No	Yes
Cystic duct entrance—distal	No	Yes
Inflammation—mild	Yes	Yes
Inflammation—marked	Yes	No
Suturing ability—poor	Yes	No
Suturing ability—good	Yes	Yes

**Table 3:** Types of dissolving solutions for the treatment of CBDS [5].

Substance	Date	Author(s)	Country
Ether	1891	Walker [135]	England
Turpentine	1908	Wright [136]	England
Chloroform	1945	Narat and Cipolla [137]	USA
Heparin saline	1971	Gardner et al. [138]	USA
Na Cholate	1972	Way et al. [139]	USA
Chenodeoxydrolic acid	1972	Danziger et al. [140]	USA
Ursodeoxycholate	1975	Makino et al. [141]	USA
Mono-octanoin	1981	Gadacz [142]	Japan
Methyl-tert-butyl ether	1985	Allen et al. [143]	USA
